# Supercritical CO_2_ Fluid Extraction and Microencapsulation of Oil from Anchovy (*Engraulis mordax*) By-Products

**DOI:** 10.17113/ftb.62.03.24.8336

**Published:** 2024-09

**Authors:** Cezar Ionuț Bichescu, Liliana Mihalcea, Raffaele Raimondo, Mihaela Cotârleț, Bogdan Păcularu-Burada, Vasilica Barbu, Gabriela Râpeanu, Gabriela Elena Bahrim, Nicoleta Stănciuc

**Affiliations:** 1 Cross-Border Faculty, Dunarea de Jos University of Galati, 111 Domnească Street, 800201 Galați, Romania; 2 Faculty of Food Science and Engineering, Dunarea de Jos University of Galati, 111 Domnească Street, 800201 Galați, Romania; 3 Mater Soc. Cons. a R. L., Brecce a Sant’Erasmo Street, 80146 Naples, Italy

**Keywords:** supercritical extraction, anchovy by-products, polyunsaturated fatty acids, transglutaminase-mediated crosslinking, binding constants

## Abstract

**Research background:**

Fish by-products are discarded as waste, which has a significant impact on the environment. They have no economic value, but there are many opportunities to turn them into high value products. Due to significant quantities generated internationally and the continuous expansion of the market for ω-3 and ω-6 fatty acids as nutraceuticals, innovative technological approaches are needed to transform this waste into marketable products with added value, while limiting the risk of environmental pollution.

**Experimental approach:**

In this study, two temperatures (40 and 60 °C) at a constant pressure during the extraction of anchovy by-products with supercritical CO_2_ fluid were used to determine extraction yield, fatty acid, tocopherol and phytosterol composition, followed by microencapsulation with two matrices based on the transglutaminase-mediated crosslinking reaction between whey protein isolates and casein. Before microencapsulation, the binding parameters were estimated using quenching studies.

**Results and conclusions:**

The results showed a higher content of total fatty acids when extracted at 40 °C, resulting in two fractions on a dry mass basis of (712±12) mg/g in the fraction obtained in the separator with code S40 and (732±10) mg/g in the fraction obtained in the separator with code S45, respectively. The monounsaturated (MUFAs) and polyunsaturated fatty acids (PUFAs) accounted for 40–44 %. The extracts showed a higher mass fraction of eicosapentaenoic acid ((28.7±1.0) mg/g) in fraction S45 when extracted at 60 °C. A minimum inhibitory and bactericidal concentration of 0.66 μg/mL against *Escherichia coli* ATCC 25922 and *Staphylococcus aureus* ATCC 25923 was found for all fractions. Higher binding constants were found for palmitoleic and oleic acids than for palmitic acid. The control variant, without crosslinking, enabled the microencapsulation of a higher amount of fatty acids, while in both powders the sum of MUFAs and PUFAs was 40 %.

**Novelty and scientific contribution:**

The approaches used in our study open up new opportunities for adding value to the fish by-products through extraction and microencapsulation, extending their potential use to food, cosmetics and nutraceuticals.

## INTRODUCTION

According to The State of World Fisheries and Aquaculture (*1*), the total production of fisheries and aquaculture reached 223.2 million tonnes in 2022, including 185.4 million tonnes of aquatic animals and 37.8 million tonnes of algae, largely due to the growth of aquaculture, particularly in Asia (*2*). In general, fisheries and fish processing industry generate significant amounts of by-products, mainly heads, skins, bones and viscera, which pose significant environmental and economic problems due to the disposal and pollution of this organic matrix (*3*). Different strategies for converting biomass into useful products have been proposed as alternatives to disposing of these by-products in the environment (*4*). For example, fish by-products can be used for different biotransformations and/or extraction of functional hydrolysates with flavouring properties (*5*), enzymes and protein hydrolysates (*6*), fertilisers, fish oil, *etc*. (*4*).

Currently, there is an unprecedented and extensive market for food supplements and nutraceuticals based on ω-3 polyunsaturated fatty acids (ω-3 PUFAs), mainly due to the growing consumer awareness of the health benefits of these bioactive substances, which have an alleviating effect on chronic diseases (*7*). Different reports have emphasised the crucial role of ω-3 PUFAs in health and well-being, as well as in the prevention of some cancers and coronary artery disease, ageing and rheumatoid arthritis, while there are reports of improving neurological functions in children (*8*, *9*). Among the ω-3 PUFAs, docosahexaenoic acid (DHA) and eicosapentaenoic acid (EPA) have been reported to have a significant impact on human nutrition (*3*, *10*).

Whole fish, fish trimmings or other fish processing by-products can be successfully used to produce fish oil enriched with ω-3 PUFAs (*2*). One of the main problems with commercial products based on ω-3 PUFAs are related to various quality issues, especially related to bioavailability and purity, while being easily oxidisable, which requires the identification of more efficient extraction methods, concentrations and formulations to obtain stable products. The interest in consumption of ω-3 PUFAs is emphasised by the recommended dose for a healthy, balanced diet of at least 0.2 g both EPA and DHA per day (*11*). However, daily EPA and DHA intake is found to be significantly lower in many Western diets. Many fish species are rich sources of ω-3 PUFAs, such as those from the *Scombridae*, *Clupeidae* and *Salmonidae* families (*7*). However, it has been reported that some fish species may contain toxic compounds such as methylmercury, polychlorinated biphenyls (PCBs), dioxins, heavy metals, *etc*. in significant amounts, so that the health-promoting effect of ω-3 intake on a daily basis can be jeopardised (*12*).

Solvent extraction, cold pressing and distillation are considered traditional methods of oil extraction, while some newer methods include microwave-assisted, ultrasound-assisted and supercritical fluid extraction (SFE). Supercritical CO_2_ fluid extraction (SFE CO_2_) has attracted considerable interest in different applications due to its ability to extract highly valuable compounds, especially non-polar compounds. The use of SFE CO_2_ brings several advantages, such as the absence of CO_2_ toxicity and non-flammability, simultaneously with the property of a green solvent. Several particularities of SFE CO_2_ are related to the use of low temperatures and the absence of oxygen, which protects the bioactive compounds, especially the thermally sensitive components, such as PUFAs (*13*). To increase the extraction yield of polar molecules, the method allows the use of co-solvents to change the polarity of the solvents (*14*). When extracted with CO_2_ fluid, different co-solvents are used to improve solvent selectivity and increase the dissolution of lipids in the solvent (generally ethanol or methanol) (*15*). The reason for the improved ability of lipids to dissolve in the solvent is due to the polar hydroxyl group attached to the ethanol molecule, while the hydrocarbon group interacts with the non-polar lipid molecules.

However, the use of fish oil in the daily diet is limited due to its taste, smell and high susceptibility to oxidation (*16*). In this context, microencapsulation becomes a unique and necessary technique, both to convert liquid fish oil into solids to facilitate transport and storage, but also to mask the unpleasant fish flavour and smell while improving the oxidative stability of the components (*17*).

Therefore, the main objective of this study is to use the SFE CO_2_ method to extract oil from anchovy by-products. Based on the data from the literature, two extraction temperatures (40 and 60 °C) at a constant pressure of 30 MPa were set, while the effect of the extraction parameters on the extraction yield, fatty acid composition, tocopherols, phytosterols and antioxidant activity was evaluated. Moreover, the antimicrobial activity of the selected oil against *Escherichia coli* ATCC 25922 and *Staphylococcus aureus* ATCC 25923 was analysed. The Rancimat method was used to determine the oxidative stability of the extracts. In addition, two biopolymeric matrices based on untreated and transglutaminase-mediated cross-linked reaction between whey protein isolates (WPI) and casein were developed and the potential of the oil to bind to these matrices was investigated based on fluorescence quenching studies. The oil was encapsulated in the developed biopolymeric matrices and the fatty acid composition, phytosterols and tocopherols, as well as the antioxidant activity of the powders were evaluated. The structure and morphology of the powders were analysed using hyperspectral dark-field microscopy. The obtained results demonstrate an original approach for the production of microencapsulated ω-3 SFE CO_2_ extracts from anchovy by-products for potential applications in innovative foods enriched with ω-3 fatty acids.

## MATERIALS AND METHODS

### Fish by-products

By-products from anchovy processing of a batch caught in August 2021 in FAO area 27, subarea IX Atlantic NE were used as fresh raw material for the extraction. The sample was provided by the company Balistreri Girolamo from lot No. P101, fished by Pescados Getaria S.R.L. on 31 August 2021. The fish by-products, consisting of heads and viscera, were preserved by freeze-drying (Alpha 1-4 LDplus; Martin Christ, Osterode am Harz, Germany) for 75 h at -42 °C and a vacuum of 100 Pa. Before extraction, the freeze-dried material was ground, vacuum-packed in batches of 500 g and stored at -18 °C.

### Reagents

Acetone, hexane, 2,2'-azino-bis(3-ethylbenzothiazoline-6-sulfonic acid) (ABTS), potassium persulphate, 6-hydroxy-2,5,7,8-tetramethylchroman-2-carboxylic acid (Trolox), ethanol, methanol, brain heart infusion (BHI) broth, agar, resazurin sodium salt, and NaCl were purchased from Sigma-Aldrich, Merck, Darmstadt, Germany. Whey protein isolate (WPI) and casein (95 % protein) were purchased from Fonterra (Clandeboye, New Zealand).

### Supercritical CO_2_ fluid extraction

The SFE CO_2_ extraction of the lipid phase from the fish by-products was carried out in a pilot extractor (Natex, Prozesstechnologie GesmbH, Ternitz, Austria) as described by Taati *et al.* (*17*), at pressure of 30 MPa and temperatures of 40 and 60 °C for 3 h in the presence of a co-solvent (10 % ethanol). The CO_2_ mass flow rate was kept constant at max. 20 kg/h. The liquid CO_2_ was cooled down to between –3 and –4 °C in the precooler, then pressurised and fed into the extractor. The extractor heating system maintained the constant temperature during extraction, thus ensuring the supercritical state of the solvent. The extracted components were fractionated with the following parameters: fraction S40 at pressure of 15 MPa and temperature of 40 °C and fraction S45 at pressure of 5 MPa and temperature of 21 °C. In each extraction batch, after depressurisation and collection of extracts, a complete recovery of the extract from the walls and the coil of the separators was achieved using a solvent mixture consisting of *V*(*n*-hexane):*V*(acetone)=3:1. After that the solvent was removed from the extracts by vacuum concentration at 40 °C (RVC 2-18 CD; Martin Christ). The extracts were coded according to the extraction temperatures as S40_40_, S45_40_, S40_60_ and S45_60_, respectively.

### The analysis of fatty acids, phytosterols and tocopherols

The method described by Mihalcea *et al.* (*18*) was used to determine the content of fatty acids, phytosterols and tocopherols of the obtained extracts and powders. In brief, the first step was to convert the fatty acids into their respective methyl esters (FAMEs) by esterification mediated by methanolic boron trifluoride. A gas chromatographic system coupled with a mass spectrometer (GC-MS) (PerkinElmer Clarus 680), equipped with an Elite-WAX capillary column (30 m×0.25 mm i.d., 0.25 µm film thickness; PerkinElmer, Shelton, CT, USA), using helium as the carrier gas (flow of 1.5 mL/min) was used for the identification of fatty acids. The FAMEs present in the extracts were quantified in selected ion recording mode (SIR), using a 5-point calibration curve prepared from 37-component FAME Mix (Supelco, Sigma-Aldrich, Merck). The phytosterols and tocopherols were determined with GC-MS using the extracts and powders dissolved in *n*-hexane. The acceptability criteria were met for all the analysed compounds at a correlation coefficient R^2^>0.99.

### Antioxidant activity

The ability of supercritical extracts to scavenge the free radicals of ABTS was assessed based by the protocol described by Lee *et al.* (*19*), with some modifications. Briefly, the fish oil was dissolved in acetone at various concentrations from 0.132 to 100 mg/mL. For the assessment of antioxidant activity of microencapsulated extracts, the microcapsules (0.25 g) were dissolved in a mixture of equal volumes of NaCl solution (10 %, *m*/*V*) and methanol. After homogenisation and ultrasonication for 30 min, 30 mL of *V*(hexane):*V*(acetone)=1:1 were added. After centrifugation (Universal 320R; Hettich, Tuttlingen, Germany) at 6000×*g* for 10 min at 4 °C, the supernatant was used for the antioxidant activity assay (*20*). A sample aliquot of 50 µL was mixed with 1.95 mL ABTS solution (*λ*_734 nm_=0.70). The absorbances of the samples were determined spectrophotometrically (Libra S22; Biochrom Ltd., Cambridge, UK) after 2 h of storage in the dark. Results were expressed as mmol of Trolox equivalents (TE) per mL based on a calibration curve with Trolox:


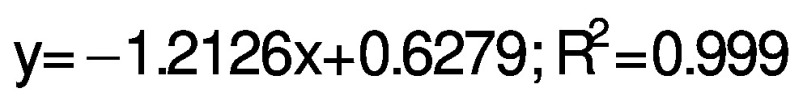
 /1/

### The evaluation of antipathogenic properties

The strains of pathogenic bacteria *Escherichia coli* ATCC 25922 and *Staphylococcus aureus* ATCC 25923 were reactivated from stock cultures in BHI broth overnight at 37 °C under aerobic conditions. The antibacterial properties of the SFE CO_2_ samples were assessed as the minimum inhibitory concentration (MIC) in 96-well microplates. A volume of 100 µL of different concentrations from 0.066 to 50 mg/mL sample were mixed with 100 µL BHI in each well, then 10 µL of bacterial inoculum (10^5^ CFU/mL) and 10 µL of resazurin (0.015 %, *m*/*V*) were added. Positive (solvent with inoculum) and negative (solvent) controls were considered. The samples were incubated for 24 h at 37 °C under aerobiosis, while the microbial activity was determined by monitoring the colour change from dark blue to pink, which indicates the bacterial growth. The minimum inhibitory concentration (MIC) was established as the lowest concentration that inhibited the growth of pathogens without a colour change of the medium (*21*). The minimum bactericidal concentration (MBC) was determined using the concentrations that inhibited the pathogens by plating 10 µL of samples from the analysed wells on BHI agar. After 24 h of incubation at 37 °C under aerobiosis, the absence of bacterial growth at a specific concentration was defined as MBC (*22*).

### Oxidative stability of fish oil extract using Rancimat analysis

The susceptibility of the fish oil extracts to oxidation was measured using the Rancimat equipment mod 892 (Metrohm LTD, Herisau, Switzerland) according to the manufacturer’s instructions. First, 3 g of fish oil extract were weighed directly into the reaction vessel. Then, the heating block was set at 80 °C, while the air flow through the reaction vessel was adjusted to 20 L/h. During oxidation, the effluent air containing volatile organic acids from the analysed extract samples was collected in the measuring vessel containing 60 mL of distilled water. The induction time (*t*_induction_/h), which characterised the oxidation stability of the oil, was the time until the secondary oxidation products were detected.

### Biopolymeric matrix design

In order to design the biopolymeric matrices for microencapsulation of the extracts, 2 g of WPI and 2 g of casein were dissolved in 100 mL of distilled water and allowed to hydrate at 4 °C for 24 h. The pH of the solutions was adjusted to 6.0, while the biopolymeric solutions were divided into two equal parts. One sample was subjected to crosslinking reaction by adding 1 mg of transglutaminase per mg protein. The enzymatic reaction was performed at 40 °C and 120*×g* for 2 h. After crosslinking, both solutions were heated at 95 °C to inactivate the enzyme and then freeze-dried (Alpha 1-4 LDplus; Martin Christ) for 48 h at -42 °C and a vacuum of 10 Pa. The powders were collected and packed in dark glass tubes. The powders were coded as C (control) and T (transglutaminase-mediated crosslinking variant). Both powders were used in the quenching and microencapsulation experiments.

### Fluorescence spectroscopy quenching studies

Fractions S40_60_ and S45_60_ obtained from the SFE CO_2_ extraction performed at 60 °C and 300 MPa were mixed in a ratio of 1:1 and further used in quenching and microencapsulation studies. The freeze-dried powders were dissolved in Tris-HCl buffer (0.1 M, pH=7.5) at a concentration of 1 mg/mL. In a typical quenching experiment, 100 µL of solution were suspended in 3 mL of Tris-HCl buffer (pH=7.5), followed by successive titration of SFE CO_2_ extracts with volumes variyng from 0 up to 0.1 mL. The samples were vigorously shaken and the fluorescence emission spectrum was recorded at the excitation wavelength of 295 nm, while data for the fluorescence intensity (AU) were collected in the wavelength range of 310-420 nm. The emission and excitation slit sizes were set to 10 nm. The quenching parameters, in terms of Stern-Volmer constants, binding constants (*K_a_*) and the number of binding sites were calculated according to Dumitraşcu *et al.* (*23*).

### Microencapsulation of oil extracts of fish by-products

The biopolymeric matrices (C_0_ and T_0_) were used to microencapsulate the extracts of fish by-products obtained using SFE CO_2_ by complex coacervation and freeze-drying. For microencapsulation, the fractioned extracts were mixed in equal parts and 2 % was added to formulated matrices, followed by homogenisation using an Ultra Turrax mixer (IKA T18 basic; IKA, Staufen, Germany) at 5000×*g* for 10 min to form a coarse emulsion. The samples were coded C_0_ and T_0_. The pH of both samples was lowered to 3.75 with 1 M HCl solution at (40±1) °C and stored at 4–6 °C overnight to enable decantation. The coacervates were freeze-dried (Alpha 1-4 LDplus; Martin Christ) at -42 °C under the pressure of 10 Pa for 48 h. The powders were then collected, packed in dark glass tubes and kept in the refrigerator at 4 °C until further analysis. The microencapsulated powders were coded as C_m_ and T_m_.

### Hyperspectral dark-field microscopy analysis

Hyperspectral dark-field microscopy (HDFM) analysis was carried out using the CytoViva hyperspectral imaging system (CytoViva Inc., Auburn, AL, USA). The microscope system (BX51; Olympus, Taunton, MA, USA) equipped with a 150 W halogen light source (Fiber-Lite, Dolan-Jenner, Boxborough, MA, USA) and a hyperspectral camera (CytoViva hyperspectral imaging system 1.4. with integrated ENVI 4.8 software) was used to generate the dark-field hyperspectral images (DF-HSI) and hyperspectral profile using 60× objective lenses. DF-HSI were processed with ENVI data analysis software (ENVI 4.8).

### Statistical analysis

The reported data are average values of triplicate measurements followed by standard deviations. Statistical analysis was carried out with Minitab 19 software (*24*), using the analysis of variance (ANOVA) and Tukey’s *post-hoc* test for 95 % confidence level (p<0.05).

## RESULTS AND DISCUSSION

### SFE CO_2_ extraction yield

It is well known that the density of a supercritical fluid is extremely sensitive to minor changes in temperature and pressure near the critical point. Therefore, in the supercritical CO_2_ fluid extraction (SFE CO_2_) experiments, a direct correlation between the temperature and CO_2_ density was observed, leading to the "crossing" of the solubility isotherms (*25*). At constant extraction pressure, the solubility of the bioactive compounds in the extracts is improved by increasing the temperature, while their diffusivity increases as the mass transfer intensifies. Consequently, a higher extraction yield of approx. 8 % was achieved at 60 °C compared to 6 % when extracted at 40 °C. Increasing the extraction temperature also has an effect on the fluidity of the lipid phase, leading to more efficient extraction (*26*). Rodrigues *et al.* (*27*) observed similar effects of temperature at constant pressure on extraction yield. These authors used SFE CO_2_ to extract oil from sardine by-products (heads and viscera) and obtained extraction yields on dry mass basis of (21.16±0.46) and (24.36±0.53) g/100, using a temperature of 35 °C at a *ρ*(CO_2_)=0.93 g/mL and 75 °C at a *ρ*(CO_2_)=0.91 g/mL, respectively, as extraction parameters. Similarly, Uddin *et al.* (*28*) investigated the effects of temperature (35–45 °C) and pressure (15–25 MPa) on oil extraction from squid viscera and obtained the highest lipid phase yield (30 %) at 45 °C, 25 MPa and an extraction time of 2.5 h. Park *et al.* (*29*) used SFE CO_2_ to extract the lipid phase from mackerel viscera and reported a lower yield at an extraction temperature of 35 °C and a pressure of 25 MPa, which increased from 13 to 16 % when increasing extraction temperature up to 45 °C.

### Fatty acid profile of the extracts

Table 1 shows the phytochemical profile of the extracts, in terms of fatty acids, phytosterols and tocopherols. It can be seen that SFE CO_2_ extraction at 40 °C allowed to obtain two fractions with a total fatty acid mass fraction on dry mass basis of (712±12) mg/g in S40_40_ and (658.2±8.4) mg/g in S45_40_, while at 60 °C, the two corresponding fractions had a total fatty acid mass fraction of (732±10) mg/g in S40 and (630±35) mg/g in S45. The saturated fatty acids (SFA) accounted for 56 % of total fatty acids in each fraction. An increase in the SFA content up to 59 % was observed in fraction S45_60_. The monounsaturated fatty acids (MUFAs) and polyunsaturated fatty acids (PUFAs) accounted for 44 %, while MUFAs were represented by oleic and palmitoleic acids. Quantitatively, all extracts had significant amounts of stearic and myristic acid, which accounted for 11–13 %. It should be noted that all extracts had significant mass fractions of eicosenoic acid, increasing with the decrease of temperature, ranging between (12.7±0.6) mg/g in S45_60_ and (24.1±0.2) mg/g in S40_40_. The lower mass fraction of eicosapentaenoic acid was obtained in S40_40_ (24.60±0.06), almost the same for S45_40_ (26.4±0.2) and S40_60_ (26.8±0.5) mg/g and the highest in S45_60_ (28.7±1.0) (Table 1).

**Table 1:** The mass fractions of bioactive compounds on dry mass basis of extracts obtained by supercritical CO_2_ fluid extraction (SFE CO_2_) of anchovy by-products

	SFE CO_2_ fraction				
Compound	S40 (*t*=40 °C)	S45 (*t*=40 °C)	S40 (*t*=60 °C)	S45 (*t*=60 °C)	
	*w*(bioactive compound)/(mg/g)				
Fatty acid					
Palmitic	(212.8±2.4)^aB^	(200.3±2.1)^aB^	(215.6±1.1)^aA^	(202.8±7.1)^aB^	
Oleic	(136.1±1.6)^bB^	(121.9±0.9)^bC^	(139.9±1.2)^bA^	(112.4±4.8)^bD^	
Palmitoleic	(104.3±1.0)^cB^	(94.7±0.5)^cC^	(110.9±1.0)^cA^	(85.8±4.2)^cD^	
Stearic	(84.7±0.5)^dB^	(77.0±0.6)^dC^	(90.6±0.8)^dA^	(80.9±3.4)^cBC^	
Myristic	(80.6±1.9)^eB^	(77.7±1.0)^dB^	(83.3±1.8)^eA^	(71.0±2.1)^dC^	
Pentadecanoic	(20.6±0.2)^gB^	(19.0±0.1)^fB^	(21.9±0.2)^gA^	(17.0±0.9)^fC^	
Linoleic	(13.8±0.1)^hA^	(11.94±0.07)^gB^	(13.8±0.2)^hA^	(10.7±0.5)^fgC^	
α-Linolenic	(5.19±0.04)^iA^	(4.43±0.03)^hB^	(4.98±0.04)^iA^	(3.9±0.2)^ghC^	
γ-Linolenic	(0.601±0.001)^jB^	(0.550±0.003)^iC^	(0.594±0.002)^jA^	(0.54±0.01)^hC^	
Eicosenoic	(24.1±0.2)^fA^	(20.4±0.2)^fB^	(19.7±0.2)^gB^	(12.7±0.6)^fC^	
Eicosapentaenoic	(24.60±0.06)^fC^	(26.4±0.2)^eB^	(26.8±0.4)^fB^	(28.7±1.0)^eA^	
Eicosadienoic	(2.921±0.004)^ijA^	(2.52±0.01)^hiC^	(2.63±0.03)^jB^	(2.03±0.07)^hD^	
Eicosatrienoic	(0.845±0.001)^jA^	(0.78±0.01)^iB^	(0.807±0.004)^jA^	(0.742±0.006)^hC^	
Arachidonic	(0.683±0.008)^jA^	(0.67±0.03)^iA^	(0.621±0.002)^jAB^	(0.60±0.01)^hB^	
Total	(712±12)^B^	(658.2±8.4)^BC^	(732±10)^A^	(630±35)^C^	
Phytosterol					
Campesterol	(0.197±0.008)^bD^	(0.273±0.006)^bC^	(0.343±0.005)^cA^	(0.303±0.006)^bB^
β-Sitosterol	(0.12±0.02)^bC^	(0.32±0.09)^bB^	(0.610±0.002)^bcA^	(0.43±0.01)^bAB^
Cholesterol	(161.0±2.1)^aD^	(215.6±3.6)^aC^	(274.6±1.9)^aA^	(250.8±7.8)^aB^
Cholesta-3,5-dien-7-one	(0.90±0.05)^bC^	(1.07±0.04)^bC^	(2.66±0.04)^bB^	(2.9±0.2)^bA^
Isofucosterol	(1.12±0.02)^bD^	(1.61±0.03)^bC^	(2.14±0.01)^bcA^	(1.88±0.01)^bB^
Desmosterol	(0.52±0.01)^bD^	(0.552±0.006)^bC^	(0.731±0.008)^bcA^	(0.67±0.02)^bB^
Total	(163.9±3.2)^D^	(219.4±5.4)^C^	(281.1±2.8)^A^	(257±11)^B^
Tocopherol					
α-Tocopherol	(1.13±0.06)^A^	nd	nd	nd

Results are presented as mean value±standard deviation. Different lower case letters in a column denote significant differences between the compounds from the same sample; different upper case letters in a row denote significant differences for the same compound from different samples using Tukey’s test (p<0.05). nd=not determined

The representative phytosterol in all fractions was cholesterol, with mass fractions varying from (161.0±2.1) mg/g in S40_40_ to (274.6±1.9) mg/g in the corresponding S40_60_ fraction. It can be seen from Table 1 that fractions S40_60_ and S45_60_ contain the highest mass fraction of campesterol of (0.343±0.005) and (0.303±0.006) mg/g, respectively. The same trend was observed for β-sitosterol, with concentrations of (0.610±0.002) and (0.43±0.01) mg/g in S40_60_ and S45_60_, respectively. Moreover, all extracts contain cholesta-3,5-dien-7-one and isofucosterol, with the highest mass fraction in S45_60_ and S40_60_, respectively. As for tocopherols, only α-tocopherol was detected in fraction S40_40_ ((1.13±0.06) mg/g).

Therefore, the profile of the extracts highly depended on temperature, pressure, extraction time, solvent flow rate, particle size, water content and the use of co-solvents. However, as it can be seen in Table 1, overall, similar mass fraction of fatty acids was extracted at both tested temperatures, while the mass fraction of phytosterols was higher when extracted at 60 °C.

Karsli *et al.* (*30*) studied the fatty acid composition of 15 commercial fish oil supplements and reported that the predominant fatty acids among the SFAs in all fish oil supplements were myristic, palmitic and stearic acids, while the content of eicosapentaenoic acid ranged from 3.51 to 20.51 %. These authors found that docosahexaenoic acid content ranged from 3.28 to 52.42 %. In the fish oil fraction extracted in this study, docosahexaenoic acid was not detected.

### Antioxidant activity of the extracts

The ABTS radical scavenging activity of the SFE CO_2_ extracts was different based on the parameters used for the supercritical extraction. Consequently, the antioxidant potential varied between *c*(TE)=0.20 and 0.29 mmol/mL; the highest value was obtained in S45 fraction extracted at 60 °C (0.29 mmol/mL). In general, the antioxidant activity of fish oil is given by the mass fraction of eicosapentaenoic (EPA) and doxosahexaenoic (DHA) acids. Therefore, the increased antioxidant activity of the extracts may be due to the different mass fraction of EPA, ranging from (24.60±0.06) mg/g in S40_40_ to (28.74±1.04) in S45_60_. The antiradical potential increased when fish oil and shrimp oil were mixed to obtain 85 % inhibition ratio for ABTS (*31*).

### Antimicrobial potential of the extracts

The fish oil extracts were characterised by their antipathogenic properties against strains of *Escherichia coli* and *Staphylococcus aureus*. Both inhibitory and bactericidal effects were observed at a concentration of 66 µg/mL of all analysed samples. Moreover, the SFE CO_2_ parameters affected the fatty acid composition of the samples, but no difference was observed in the antibacterial properties. The experimental data are comparable to the results reported by Cardeira *et al.* (*32*), demonstrating that the antipathogenic potential of the supercritical fluid extracts of sardine by-products can vary between 70 and 1.56 mg/mL, depending on the extraction parameters. Thus, higher concentrations of the extracts of the sardine by-products were determined to have a bactericidal effect on *Staphylococcus aureus* ranging from 9.38 to 68.75 mg/mL, while Azmi *et al.* (*33*) combined the fish oil with lemon essential oil to suppress the growth of *Escherichia coli* strain at 250 mg/mL.

Huang *et al.* (*34*) reported that ω-3 PUFA showed antimicrobial activity against various oral pathogens, including *S. mutans, C. albicans, A. actinomycetemcomitans*, *F. nucleatum* and *P. gingivalis.* These authors suggested that linoleic acid, γ-linolenic acid, arachidonic acid, palmitoleic acid and oleic acid were responsible for this activity. Therefore, given the prevalence of palmitic, oleic and palmitoleic acids in all the extracted fractions, the antimicrobial activity of the extracts against *Escherichia coli* and *Staphylococcus aureus* may be due to the high concentration of these fatty acids, while their cumulative effect should not be excluded.

### Oxidative stability of the extracts

The oil with high content of MUFA and PUFA is prone to oxidation, which consists of three successive stages, namely initiation, propagation and termination. In general, the presence of oxygen initiates the oxidative process by reacting with hydrogen atom from the double bonds found in MUFA and PUFA, which results in the formation of highly reactive free radicals with unpaired electrons. The free radicals seek to lose electrons in order to become stable, trying to form paired states with the nearby fatty acids, thus forming new radicals. The chain reaction persists until two radicals combine and thus neutralise their reactivity (*35*). Oxidative alterations lead to significant changes in oil, such as rancidity, generation of free fatty acids, nutrient loss, undesirable odorus and flavorus, and appearance of toxic compounds. The undesirable secondary metabolites are represented by conjugated dienes, aldehydes, peroxides and polar compounds (*36*).

The induction time, defined as the time corresponding to the inflection point of the conductivity *vs* time, was used for quantitative description of the oxidative stability of the oil extracts from fish by-products. The induction time of 3 g of fish oil extract varied from (0.010±0.001) to (0.81±0.04) h at a temperature of 80 °C and an air flow of 20 L/h (data not shown). The S45 fraction obtained at 60 °C was significantly more stable against oxidation ((0.81±0.04) h).

García-Moreno *et al.* (*37*) reported an oxidation stability index of 1.61 h, determined by the response surface method, for 6.91 g of partially purified cod liver oil at an air flow of 25 L/h and a temperature of 88.26 °C. Méndez *et al.* (*38*) suggested an induction time of 3.2 h for anchovy oil, 1.4 h for hake liver oil and 1.5 h for sardine oil at an air flow of 20 L/h and a temperature of 80 °C for 3 g of partially purified oil.

### Fluorescence quenching studies

**Fig. 1:**
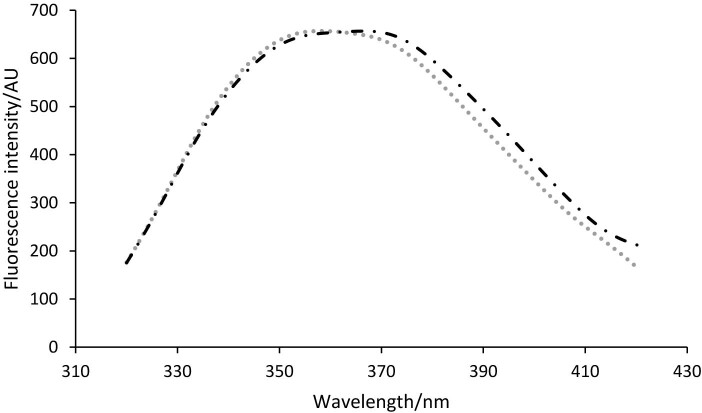
Fluorecence emission spectra of the isolated whey protein and casein matrices without (C_0_) (grey dotted line) and with (T_0_) transglutaminase-mediated crosslinking (black dotted line)

Prior to microencapsulation, the fluorescence quenching studies were used to evaluate the potential of the fatty acids to bind to the biopolymeric matrices based on WPI and casein without (C_0_) and with (T_0_) transglutaminase-mediated crosslinking. Fig. 1 shows the fluorescence emission spectra of the two matrices.

Fig. 1 shows that the maximum emission wavelength (*λ*_max_) for sample C_0_ was recorded at 358 nm, while crosslinking led to a significant 8.5-nm redshift up to 366 nm. The redshift is due to the dipole-dipole interactions resulting from crosslinking. Consequently, the *λ*_max_ redshift implies the displacement of the hydrophobic regions in the more polar environment, which allows the molecules to unfold and to bind different ligands from the extracts. Moreover, Taheri-Kafrani *et al.* (*39*) suggested that the redshift in wavelength and the increase in the fluorescence intensity at the *λ*_max_ is due to the Trp transfer to a more hydrophilic environment and the increase in polar interactions.

In addition, the interactions between biopolymeric matrices and ligands were studied by successive titration with fractions S40_60_ and S45_60_, respectively, by increasing the volume of the extracts from 0 to 100 μL. The fluorescence quenching effect could be observed in both samples. Therefore, with the increased volume of the extract, the fluorescence intensity of the protein solutions gradually decreased, with a higher quenching effect in the case of crosslinked matrices (T_0_). When titrating with fraction S40_60_, the quenching effect reached a maximum of ~47 % with sample T_0_ compared to ~39 % with sample C_0_, while in quenching studies with S45_60_ a maximum effect was found in sample C_0_ (~43 %) compared to 41 % in sample T_0_. The successive titration with the two extracts caused significant 8.5-nm redshift in sample C_0_ and 2.5-nm shift in sample T_0_. These results indicate a higher molecular flexibility in C_0_ than structural rigidity due to the crosslinking in T_0_. Therefore, the addition of the extract T_0_ allowed to preserve the crosslinked structure, causing only rearrangements of the hydrophobic subdomain to which the tryptophan residues belong. The binding constants (*K*_a_) and number of binding sites (*N*) were calculated for the predominant fatty acids from the extracts and are shown in Table 2.

**Table 2:** The binding constant (*K*_a_) values for the fractions (S40 and S45) extracted with supercritical CO_2_ fluid extraction with isolated whey protein and casein matrices without (C_0_) and with (T_0_) transglutaminase-mediated crosslinking

	C_0_		T_0_	
Fatty acid	S40	S45	S40	S45
	*K*_a_/(·10^3^ L/mol)			
Palmitic	(19.2±0.2)^cA^	(17.0±0.85^cBC^	(15.8±0.3)^cC^	(18.2±0.5)^cAB^
Palmitoleic	(40.4±0.4)^aB^	(38.7±0.5)^aC^	(32.6±0.5)^aD^	(43.6±0.4)^aA^
Oleic	(37.2±0.7)^bA^	(29.7±0.4)^bC^	(25.7±0.2)^bD^	(33.8±0.5)^bB^

Results are presented as mean value±standard deviation. Different lower case letters in a column denote significant differences between the *K*_a_ of the same sample for different fatty acids; different upper case letters in a row denote significant differences for the same *K*_a_ and fatty acid within different samples using Tukey’s test (p<0.05). The extract used for quenching experiments was a mixture of fractions *m*(S40):*m*(S45)=1:1 obtained from the extraction at 60 °C and 300 MPa

The data in Table 2 show that palmitic, palmitoleic and oleic acids had a single class of binding sites on both C_0_ and T_0_. Different values of *K*_a_ were estimated for fractions S40_60_ and S45_60_. For example, the *K*_a_ value for the binding of palmitic acid was higher when fraction S40_60_ was bound to C_0_ ((19.2±0.2)·10^3^ L/mol) than when fraction S45_60_ was bound to T_0_ ((18.2±0.5)·10^3^ L/mol). The same trend was observed for palmitoleic acid, with higher *K*_a_ values of (40.4±0.4)·10^3^ L/mol and (43.6±0.4)·10^3^ L/mol, respectively. Given the predominance of β-lactoglobulin in WPI, Simion (Ciuciu) *et al.* (*40*) proposed the presence of two lipophilic ligand binding sites in the protein: a central hydrophobic β-barrel and a superficial pocket. It can be assumed that fatty acids bind to the superficial pocket, while other molecules bind to the calyx due to the intrinsic flexibility of the conjugates. Therefore, it can be assumed that the WPI and casein complexes act as protein-based multiligand carriers that enable the binding of small molecules.

### Microencapsulation of the fish oil obtained with supercritical extraction

Table 3 shows the phytochemical profile of microencapsulated powders (C_m_ and T_m_). It can be observed that C_m_ allowed the microencapsulation of a higher amount of fatty acids and phytosterols, especially palmitic, palmitoleic, myristic and stearic acids and cholesterol. Total fatty acid mass fraction of (81.2±0.9) and (68.8±0.8) mg/g was found in C_m_ and T_m_, respectively. The main fatty acids in both powders were saturated fatty acids (SAT), representing 60 % of the total, while MUFAs and PUFAs accounted for 40 %. The sample C_m_ contained (11.1±0.1) mg/g PUFAs, from which ω-3 were predominant (60 %), followed by ω-6 (39 %) and ω-11 (26 %). The PUFA mass fraction in T_m_ was (9.6±0.6) mg/g, from which ω-3 were predominant (48 %), followed by ω-6 (31 %) and ω-11 (21 %). However, the T_m_ sample had a higher mass fraction of α-tocopherol.

**Table 3:** The mass fractions of bioactive compounds on dry mass basis of the extracts from anchovy by-products obtained by supercritical CO_2_ fluid extraction (SFE CO_2_) microencapsulated in the isolated whey protein and casein matrices without (C_m_ and with transglutaminase-mediated crosslinking (T_m_)

		Powder			
Compound		C_m_		T_m_	
		*w*(compound)/(mg/g)			
Fatty acid					
Palmitic		(32.7±0.1)^aA^		(27.82±0.06)^aB^	
Oleic		(10.97±0.03)^bA^		(9.2±0.2)^bB^	
Palmitoleic		(9.39±0.07)^cA^		(7.85±0.05)^cB^	
Stearic		(7.15±0.04)^eA^		(6.1±0.1)^eB^	
Myristic		(8.4±0.2)^dA^		(6.96±0.02)^dB^	
Pentadecanoic		(1.18±0.01)^iA^		(1.002±0.005)^iB^	
Linoleic		(3.83±0.03)^fA^		(3.16±0.05)^fB^	
α-Linolenic		(0.891±0.005)^jA^		(0.81±0.01)^iB^	
γ-Linolenic		(0.35±0.01)^lA^		(0.35±0.01)^kA^	
Eicosenoic		(2.93±0.03)^hA^		(2.42±0.04)^hB^	
Eicosapentaenoic		(2.30±0.01)^gA^		(2.01±0.03)^gB^	
Eicosadienoic		(2.923±0.004)^kA^		(2.52±0.01)^jB^	
Eicosatrienoic		(0.594±0.006)^klA^		(0.58±0.01)^jA^	
Arachidonic		nd		nd	
Total		(81.2±0.9)^A^		(68.8±0.8)^B^	
Phytosterol					
Campesterol		nd		nd	
β-Sitosterol		nd		nd	
Cholesterol		(19.3±1.5)^aA^		(16.6±0.4)^aB^	
Cholesta-3,5-dien-7-one		(0.20±0.02)^bA^		(0.18±0.01)^bB^	
Isofucosterol		(0.05±0.01)^cA^		(0.030±0.003)^dB^	
Desmosterol		(0.05±0.01)^cA^		(0.040±0.005)^cA^	
Total		(19.6±2.2)^A^		(16.8±0.6)^B^	
Tocopherol					
α-Tocopherol	(0.044±0.005)^B^		(0.055±0.006)^A^	

Results are presented as mean value±standard deviation. Different lower case letters in a column denote significant differences between the compounds from the same sample; different upper case letters in a row denote significant differences for the same compound from different samples using Tukey’s test (p<0.05). The extract used for microencapsulation experiments was a mixture of fractions *m*(S40):*m*(S45)=1:1 obtained from the extraction at 60 °C and 300 MPa. nd=not determined

Sànchez *et al.* (*41*) encapsulated krill oil in gum Arabic in a ratio of 1:4 and reported that the spray-dried microcapsules contained 21 % of eicosapentaenoic and docosahexaenoic acid of the total fatty acids, with low ω-6/ω-3 and SAT/unsaturated fatty acid ratios. In our study, the SAT/MUFA ratio was 1:2.38 and 1:2.41 in C_m_ and T_m_, respectively, while SAT/PUFA ratio was 1:4.4 in C_m_ and 1:6.36 in T_m_.

The antioxidant activity (ABTS) of the microencapsulated powders was in the range *b*(TE)=0.69–4.13 mmol/g, with the highest value for T_m_ of (4.13±0.03) mg/g. Our results confirm the findings of similar studies from the literature that involved transglutaminase, inulin and soy protein isolate to improve the microencapsulation efficiency of fish oil (*42*). Fan *et al.* (*43*) showed that the conjugated menhaden oil with epigallocatechin gallate, whey protein isolate and genipin improved the ABTS scavenging activity, leading to a decrease of the IC_50_ values for ABTS, from more than 2000 µg/mL for the native whey protein isolate, up to 83.50 µg/mL for the crosslinked microcapsules.

### Dark-field hyperspectral images and hyperspectral profile of the powders

**Fig. 2:**
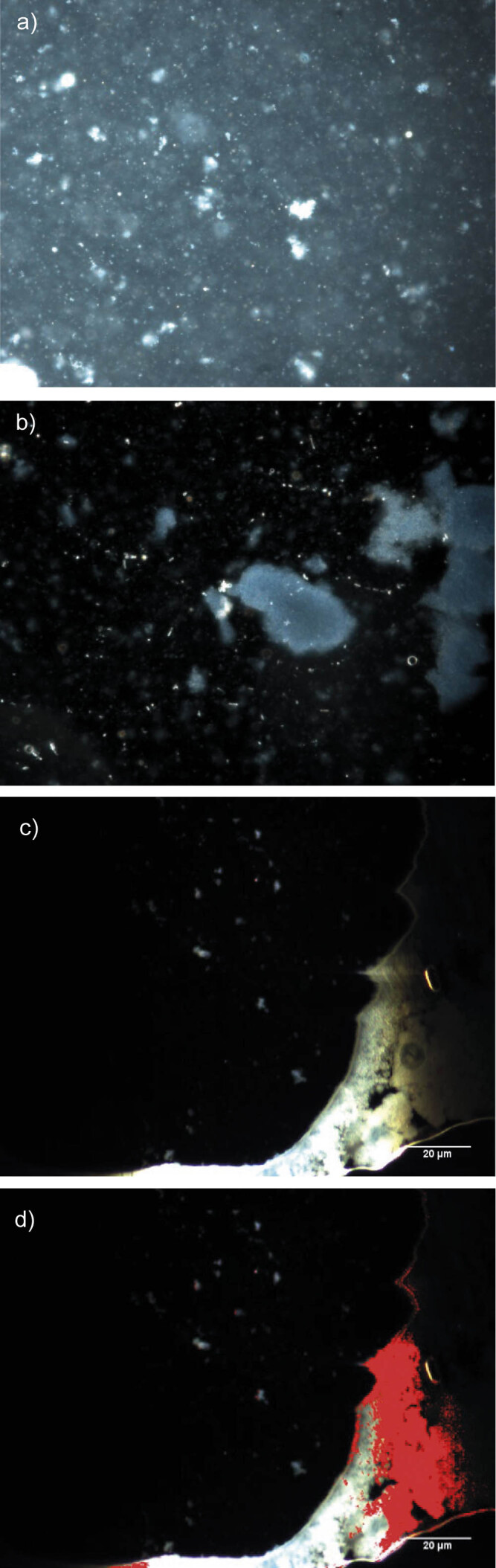
Dark-field hyperspectral images (DF-HSI) of the isolated whey protein and casein matrices: a) without (C_0_) and b) with transglutaminase-mediated crosslinking (T_0_), and microencapsulated extract of anchovy by-products: c) without (C_m_) and d) with transglutaminase-mediated crosslinking (T_m_)

**Fig. 3:**
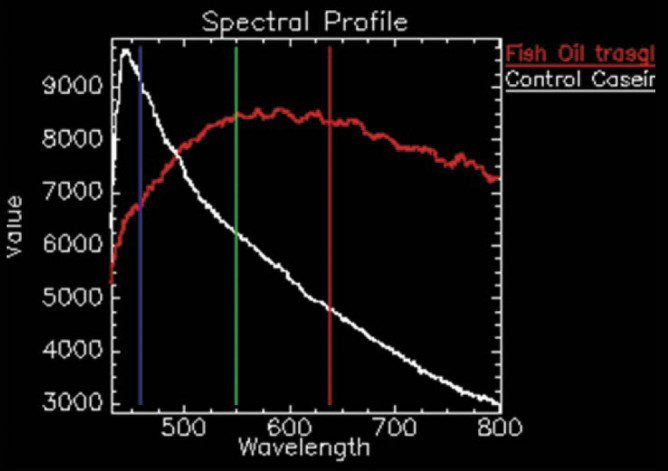
Mapping of the spectral profiles of control sample (T_0_; white) *vs* microencapsulated fish oil sample (T_m_; red) using CytoViva HSI optical microscopy

The microscopic details of the microencapsulated powders are shown in Fig. 2. The crosslinking reaction mediated by transglutaminase (T_0_) causes aggregation/agglutination of the protein matrices (WPI and casein) so that irregular formations appear, larger than those in the control sample (C_0_). The encapsulation pattern of the lipid component in the C is in the form of irregular scales with dimensions between 10 and 50 μm. Xiong *et al.* (*44*) suggested the presence of some raisin-like structures due to a low ratio between the protein shell and the lipid core. It is likely that the ratio between the lower protein content of the matrix and the temperatures near freeze-drying causes the shrinkage of the micelles and the appearance of irregular shapes. Spectral mapping showed in red all the unique elements in T_m_, with the fish oil trapped in the biopolymer matrix. The mean spectral comparison of T_m_ *vs* T_0_ is shown in Fig. 3.

An inversion of the spectra is evident. If the polymer matrix generates a peak at 450 nm, the presence of fatty acids from the fish oil inverts the curve and generates an absorption maximum between 550 and 650 nm, probably due to the interactions between the components of the fish oil and the proteins during microencapsulation (*44*).

## CONCLUSIONS

In this study, supercritical CO_2_ extraction of oil from anchovy by-products was tested to obtain extracts with a different phytochemical profile depending on the extraction parameters. The results showed an increased extraction yield when the oil was extracted at a temperature of 60 °C and a pressure of 30 MPa. In all fractions, saturated fatty acids represented the major fractions, while monounsaturated and polyunsaturated fatty acids accounted for more than 40 %. The predominant saturated fatty acids were palmitic acid, stearic acid and myristic acid, while the monounsaturated fatty acids were represented by oleic acid and palmitoleic acid. Polyunsaturated fatty acids accounted for up to 10 %, represented by eicosenoic acid and eicosapentaenoic acid. The extracts showed a minimum inhibitory and bactericidal concentration of 0.66 μg/mL, with the higher oxidative stability observed for the S45 fraction extracted at 60 °C. From a microencapsulation perspective, two binding matrices were designed based on non-treated and transglutaminase-mediated crosslinking between whey protein isolates and casein. Based on the quenching potential of the extracts, higher values for the *K*_a_ of palmitoleic and oleic acids were observed than of palmitic and oleic acids. Both matrices successfully encapsulated extracts from anchovy by-products, resulting in powders with a higher total fatty acid content in the non-crosslinked variant. In both powders, monounsaturated and polyunsaturated fatty acids accounted for 40 %. The obtained results highlight the underutilised potential of by-products generated during the industrial processing of fish, with the aim of creating solutions to improve the functional properties of food while offering innovative solutions for environmental protection by integrating valuable compounds into different matrices. Further studies are needed to increase the yield of ω-3 and ω-6, while some other microencapsulation matrices should be tested to improve the potential applications in food or nutraceuticals. In addition, further studies should be conducted to evaluate the seasonal variation in nutrients and functional compounds with the aim of determining the most favourable season for processing and extracting bioactive compounds from anchovy by-products.
